# Intestinal Helminthiasis in Children of Gurez Valley of Jammu and Kashmir State, India

**DOI:** 10.4103/0974-777X.62872

**Published:** 2010

**Authors:** Showkat Ahmad Wani, Fayaz Ahmad, Showkat Ali Zargar, Ayesha Amin, Zubair Ahmad Dar, Pervaiz Ahmad Dar

**Affiliations:** *P. G. Department of Zoology, The University of Kashmir, Srinagar, Kashmir*; 1 *Department of Gastroenterology, SK Institute of Medical Sciences, Srinagar, Kashmir*

**Keywords:** Children, Gurez valley, Helminth infection, Prevalence rates

## Abstract

**Introduction:**

This paper is a part of the helminthological studies carried out on school-going children of the Kashmir Valley and deals with the status of intestinal helminths in the children of Gurez Valley and to assess epidemiological factors associated with the extent of endemic disease so that control measures are adopted.

**Material and Methods:**

Stool samples were collected from 352 children from Gurez Valley. The samples were processed using Kato-Katz thick smear technique, and microscopically examined for intestinal parasites.

**Results:**

Of the 352 children surveyed, 75.28% had one or more types of intestinal helminthes. Prevalence of *Ascaris lumbricoides* was highest (71.18%), followed by *Trichuris trichiura* (26.42%), *Enterobius vermicularis* (13.92) and *Taenia saginata* (5.39%). Conditions most frequently associated with infection included the water source, defecation site, personal hygiene, and the extent of maternal education.

**Conclusion:**

The study shows a relatively high prevalence of intestinal helminths and suggests an imperative for the implementation of control measures.

## INTRODUCTION

According to WHO, the level of helminth infection can be viewed as an index of a community's progress towards a desirable level of sanitation.[1] Poor hygienic living conditions give rise to helminth infections in children, with the prevalence of such infections being an excellent indicator of socio-economic status.[2] Many helminth parasites remain major contributors to morbidity in developing countries. Among the effects associated with these parasites are growth retardation, intestinal obstruction, hepatic and biliary diseases, impaired cognitive development, and nutritional effects such as iron deficiency anemia.[3] Intestinal parasites are transmitted through the contamination of water, soil, and food by feces, a direct consequence of poor hygienic and living conditions.[4]

According to a WHO estimate, globally there are 800-1,000 million cases of ascariasis, 700-900 million cases of trichiuriasis, 200 million cases of giardiasis, and 500 million cases of amoebiasis.[5] Among the intestinal parasitic infections, helminth infections are the most common on the Indian sub-continent. There are only a few hospital-based studies on the prevalence of intestinal parasites in Kashmir,[6,7] which may not be a true indicator of the prevalence of gastrointestinal parasitic infestation since most of asymptomatic children may have been missed. This is a first attempt to know the status of intestinal helminthiasis in Gurez valley of Jammu and Kashmir State and was conducted from July 2008- October 2008.

## MATERIAL AND METHODS

Gurez Valley lies in the north of Kashmir between the Himalayan range of mountains under Indian administration. It is situated between 74°-30″ East longitude and 34°-23″ −34°-41″ latitude. It is approached from Bandipore town through Razdan pass at an altitude of 11673 feet. Climate in Gurez is naturally divided into two equal phases of six months each. From May to October climate remains pleasant, while in the remaining six months climate is uncertain and regrous, marked with heavy snowfall.[8] Official meetings with the personnel from health services and schools, as well as parents and school children from the study sites, were carried out in order to explain the protocol of the study. This study was conducted in 3 middle schools of Dawar, Budugam and Kanzalwan localities of Gurez Valley. In total, 352 children between ages of 1-15 years (mean = 9.1±2.8 years) with no disabilities or those not receiving antiparasitic treatment were included in this study. Initially, 420 children were willing to participate voluntarily, but 68 were rejected during the study because they either had contaminated fecal samples or decided not to participate. Written consents were required from both parents in order for the children to participate. Children requiring medical assistance were properly treated or referred for medical attention. With a view of maintaining age stratification, the study population was divided into three age groups, i.e., 1-5 years, 6-10, and 11-15.

Collection of the socio-economic characteristics of the children's families was undertaken with a structured questionnaire. The interviews were administered face-to-face with mothers in children's schools. The level of education of the mothers (below or above 10^th^ class), sanitation facilities, type of drinking water (tap, well, or stream/pond water) and defecation site (open or modern sanitary latrine) were collected as proxy variables of socio-economic conditions. The children's ages were obtained through school records. Study participants were provided with a labeled clean stool container containing l0 ml of 10% formalin. Toilet tissue paper and a clean piece of stick were given to collect a fresh morning stool specimen on the next day. In addition, a prepared cellotape slide for the examination of *Enterobius* infection was also provided. Every child was instructed to bring his/her own stool, so that no mixing occurred. After collecting the stool specimens, they were processed immediately using simple smear and Kato-Katz thick smear techniques. Cellotape slides were directly observed under the microscope for the *Enterobius* eggs. All the parasites recovered were recorded and descriptively analyzed. Statistical analysis was carried out by χ^2^ test.

## RESULTS

In Gurez Valley, out of 352 children surveyed, 265 (75.28%) were positive for intestinal helminths. Prevalence of *Ascaris lumbricoides* was 71.87%, followed by *Trichuris trichiura* (26.42%), *Enterobius vermicularis* (13.92%) and *Taenia saginata* (5.39%). Single species infection was seen in 38.63% of the infected children, whereas 36.64% were infected with multiple species of helminth parasites. The prevalence of infection peaked in the age group of 11-15 years (84.91%) followed by the age group of 6-10 years (81.70%) and age group of 0-5 years (50.54%) [*P*<0.05]. The differences in prevalence rates between male, female and rural urban children was insignificant (*P*>0.05). Water source, defecation site, personal hygiene and maternal education were significant risk factors in predicting the intestinal helminth infection (*P*<0.05) [Table 1].

**Table 1:** Factors associated with the prevalence of intestinal helminths

Variable	Determiner	Number	+ve	% age	χ^2^	*P* value
Age	0-5	91	46	50.54	12.49	0.003
	6-10	82	67	81.70		
	11-15	179	152	84.91		
	Total	352	265	75.28		
Gender	Male	228	178	78.07	1.87	0.1
	Female	124	87	70.16		
Residence	Rural	272	209	76.83	2.26	0.2
	Urban	80	56	70		
Water source	Tap water	295	211	71.52	11.43	0.001
	Well water	29	27	93.10		
	River/Stream	28	27	96.4		
Condition of water Boiled	103	58	56.31	10.96	0.001
	Unboiled	249	207	83.13		
Defecation site Open fields	10	9	90	11.03	0.002
	Open latrine	289	232	80.27		
	Modern latrine	53	24	45.28		
Personal hygiene	Clean nails	112	65	58.03	10.83	0.001
	Dirty nails	240	200	83.33		
Maternal education	Illiterate	259	202	77.99	5.93	0.04
	Secondary	92	63	68.47		
	Graduate	1	1	100		

## DISCUSSION

The present study indicated a relatively high prevalence (75.28%) of intestinal parasites in the schoolchildren of Gurez Valley. Studies conducted on the frequency distribution of gastrointestinal helminths by Bundy *et al*. (1988) showed a high overall prevalence of 62% among the urban slum children of Malaysia.[9] Rodriguez *et al*. (2000) reported a high prevalence of 72% among the schoolchildren studying in a public institution in Maracaibo, Venezuela[10] and Legesse and Erko (2004) also noted a high prevalence of 88.2% among the schoolchildren in rural Ethiopia[11] The high prevalence in Gurez Valley is probably a consequence of a low standard of living, poor sanitation, lack of personal hygiene, traditional methods of agriculture, indiscriminate defecation, the use of human feces as fertilizers and other occupational work. Age-specific prevalence data show a relationship between age and prevalence of parasites. The highest prevalence, i.e. 84.91%, was seen in the 11-15 age group followed by 81.70% in the 6-10 age group, and 50.54% in the 1-5yr age group. Similar age-related prevalence variations among schoolchildren have been reported by other investigators. For example, Ibrahim (2002) in Gaza, Palestine, showed that most of the positive cases were clustered in the middle age group, followed by the 8- 9-years age groups.[12] Even though gender was not a significant risk factor for prevalence of intestinal parasitic infections, males were more likely to be infected (78.07%) than females (70.16%). This finding can be partially explained by the difference in gender behavior. Males in their early age are likely to acquire work responsibilities in outdoor environments and girls are likely to commence duties indoors because of social and religious restrictions.

The outdoor environment, i.e. farmlands or playing fields, are a common place for defecation by males during working or playing hours and, therefore, contamination of soil in these areas would constitute a significant risk for parasite transmission. Singh *et al*. (1984) reported similar results in their study of a rural community in Varanasi, India, where in males exhibited a higher prevalence of intestinal parasitism than females.[13] A study conducted by Ibrahim (2002) on the prevalence of parasites among schoolchildren in Gaza, Palestine, likewise showed a significantly higher prevalence of infection among males compared to females.[12] Children who sourced drinking water from rivers or streams and wells were found to harbor a greater prevalence of infection than those who had access to tap water. This pattern of infection has been confirmed in various studies the world over.[14,15]

Curtis *et al*. (1995) demonstrated that mothers from poor communities in Burkina-faso, Africa, with access to tap water in the yard, were more likely to use safe hygiene practices than mothers using wells in the yard.[16] It is possible that poor hygiene practices associated with access to water is highly probable risk factor for increased parasitic infection among children. It is also evident from the present study that children with better personal hygiene had a lower prevalence of intestinal parasitic infections than those living in less hygienic conditions (*P*<0.01). In our study, it was also found that maternal education was a significant risk factor for the prevalence of infection, i.e., prevalence of infection decreases as the level of maternal education increases. Apparently, this factor extensively contributes to controlling risk factors for intestinal infections. Maternal education has been found to be the most important risk factor for parasitism in other studies as well.[17,18]

## CONCLUSION

The present study reveals that intestinal helminths are abundant among schoolchildren of Gurez valley. This situation strongly calls for the institution of control measures, including treatment of infected individuals, improvement of sanitation practices, provision of clean water and further studies on the abundance of intestinal protozoan infections in the children of Gurez valley. The impact of each measure would be maximized through a health education program directed at schoolchildren and their mothers in particular, and to communities in general.
